# Predictive Value of Centered Clinical Asymmetric Lower Limb Edema in Diagnosing Deep Vein Thrombosis in Puerperium

**DOI:** 10.3390/jcm14103320

**Published:** 2025-05-09

**Authors:** Catalina Filip, Daniela Roxana Matasariu, Alexandra Ursache, Cristina Furnica, Gabriel Ioan Anton, Cristiana Filip, Vasile Lucian Boiculese, Demetra Gabriela Socolov, Raluca Ozana Chistol

**Affiliations:** 1 Department of Vascular Surgery, University of Medicine and Pharmacy “Grigore T. Popa”, 700111 Iasi, Romania; catalina.filip@umfiasi.ro; 2 Department of Vascular Surgery, CHU “Gabriel Montpied”, 63000 Clermont-Ferrand, France; 3 Department of Mother and Child Medicine, University of Medicine and Pharmacy “Grigore T. Popa”, 700111 Iasi, Romania; alexandra.ursache@umfiasi.ro (A.U.); demetra.socolov@umfiasi.ro (D.G.S.); 4 Department of Obstetrics and Gynecology, Cuza Voda Hospital, 700038 Iasi, Romania; anton.gabriel-ioan@d.umfiasi.ro; 5 Department of Morpho-Functional Sciences I, University of Medicine and Pharmacy “Grigore T. Popa”, 700111 Iasi, Romaniaraluca-ozana.chistol@umfiasi.ro (R.O.C.); 6 Institute of Forensic Medicine, 700455 Iasi, Romania; 7 Department of Biochemistry, University of Medicine and Pharmacy “Grigore T. Popa”, 700111 Iasi, Romania; cristiana.filip@umfiasi.ro; 8 Biostatistics, Department of Preventive Medicine and Interdisciplinarity, University of Medicine and Pharmacy “Grigore T. Popa”, 700111 Iasi, Romania; lboiculese@gmail.com; 9 Department of Medical Imaging, “Prof. Dr. George I.M. Georgescu” Cardiovascular Diseases Institute, 700503 Iasi, Romania

**Keywords:** platelet, thrombosis, asymmetrical edema, lower limb, pregnancy, postpartum, thrombophilia, D-dimer, deep vein thrombosis

## Abstract

**Background:** Peripheral vein thrombosis during pregnancy poses serious diagnostic challenges due to the intertwining of its clinical symptoms with normal pregnancy modifications. **Methods:** We analyzed and compared the paraclinical test results of singleton pregnant women and women in the first six weeks postpartum who presented with significant lower limb inequality. **Results:** Our data revealed three predictors of deep vein thrombosis (DVT): mean platelet volume (MPV), with a one-unit increase in MPV being associated with a 1.497-fold higher risk of thrombosis (*p* = 0.008); platelet distribution width (PDW), with a one-unit decrease in PDW increasing thrombosis risk (odds ratio (OR) = 1.17, *p* = 0.003); anemia, with its presence increasing the risk of thrombosis by 8.46 times (*p* = 0.003); and fibrinogen, with a one-unit increase in its level increasing DVT risk 1.003-fold. **Conclusions:** Significant lower limb inequality might be used as a predictor of DVT during puerperium.

## 1. Introduction

Pregnancy and the postpartum period are known for their 4- to 5-fold increased risk of venous thrombosis caused by an estrogen-determined yet incompletely understood hyper-coagulation status [1,2,3]. Although it is a pregnancy protection necessity, the equilibrium of the prothrombotic state is frequently very easily disrupted by associated risk factors, such as being overweight, obesity, smoking, immobilization, hypertension, diabetes, thrombophilia, assisted reproduction interventions, personal history of thrombosis, and other pathologies [2,3,4,5,6]. Half of the clotting episodes in women of reproductive age are associated with pregnancy [7]. Approximately half of thrombotic events occur during pregnancy, while the other half occur during the short postpartum period, underlining the increased postpartum risk based on per-day estimates [4]. Venous thrombosis remains one of the major contributors to increased maternal morbidity and mortality, with long-term sequelae represented by complications such as post-thrombotic syndrome [5,8].

Around three-quarters of women with venous thrombotic events have peripheral deep vein thrombosis. Peripheral vein thrombosis in pregnancy poses serious diagnostic challenges due to the intertwining of its clinical symptoms with normal pregnancy modifications [1]. Deep vein thrombosis (DVT) often exhibits no clinical symptoms, generating additional challenges for the physician [8]. Additionally, studies in the literature suggest that a hypercoagulable state develops as early as the first trimester of pregnancy. This condition arises due to increased pro-coagulation factors, reduced fibrinolytic activity, and the onset of venous stasis caused by progesterone-induced decreased venous tone, occurring even before the compression of the inferior vena cava by the growing uterus. Endothelial dysfunction further contributes to this process, completing Virchow’s triad [1,2,3,4].

The acknowledgment that DVT is the most frequent form of thrombotic event occurring during pregnancy and that the usual thrombotic site is in the proximal lower-left extremity offers the clinician minimal help with managing cases with increased DVT suspicion. The exact mechanism underlying this predominance is partially but incompletely justified by the location of the common left iliac vein between the lumbar vertebras and the homolog artery [4,7,9]. In 2022, Raia-Barjat et al. identified seven scores for assessing the risk of venous thrombotic events [10], from which Lindqvist et al. studied the risk of venous thrombosis during puerperium [11,12]. Subsequently, there were many attempts to elaborate, validate, and later apply these scores in clinical practice. Facing difficulties associated with low incidences of venous thromboembolism (VTE) and variations in the risk factors considered, few of these scores proved effective in the puerperium, and there is still no consensus standardized risk score for venous thromboembolism [10,13,14].

Clinical examination alone is largely unreliable in detecting DVT, particularly during the puerperium, with diagnostic confirmation occurring in only approximately one-third of suspected cases. However, it remains valuable because compression ultrasonography’s primary non-invasive diagnostic tool’s accuracy is significantly enhanced when preceded by a clinical probability assessment strongly suggesting DVT. DVT should be suspected in patients presenting with one or more of the following symptoms: calf pain, calf tenderness, edema, increased local temperature, superficial venous dilatation, and, in cases of severe obstruction, cyanosis [8,15,16,17].

Given the variability in results in the existing literature, our study aimed to evaluate the accuracy of lower limb edema in detecting DVT in pregnant and postpartum women (within six weeks after delivery), in association with blood tests and major risk factor assessment, compared with compression ultrasound Doppler evaluation. A reliable clinical examination can be highly valuable in less developed and developing countries where diagnostic resources are scarce and access to specialized care is limited. By utilizing universally accessible methods, an accurate clinical assessment can help reduce healthcare costs and ensure that only patients with a genuine need for further evaluation are referred for specialized investigations. Additionally, due to the physiological occurrence of lower limb edema during pregnancy, repeated compression ultrasonography to rule out DVT can be financially burdensome when clinical suspicion arises repeatedly. Moreover, minimizing unnecessary investigations can help alleviate patient anxiety and reduce the psychological burden associated with suspected DVT.

## 2. Materials and Methods

This study included pregnant women and women in the first six weeks of postpartum who were hospitalized at the obstetrics and gynecology “Cuza Voda” Maternity Hospital, the “Saint Spiridon” Clinical Hospital, and the “Dr. C. I. Parhon” Clinical Hospital in Iasi, Romania, between September 2015 and September 2024. This study was conducted in accordance with the Declaration of Helsinki and was approved by the Ethics Committee of the University of Medicine and Pharmacy “Grigore T. Popa”, Iasi (105/22 July 2021), and of the Obstetrics and Gynecology Hospital “Cuza-Voda”, Iasi, Romania (10426/24 August 2021). Informed consent was obtained from all subjects involved in this study.

We included singleton pregnant women (between 5 and 39 weeks of gestation), as well as women in the first six weeks postpartum who presented with significant lower limb inequality. We chose this timeframe because severe thrombotic events occur more frequently during this period. The calves of these women were measured with a tape measure 10 cm (cm) below the tuberosity of the tibia, and a difference of more than 3 cm between both calves was considered significant and noted as lower limb inequality [8]. The hip circumference was measured at its widest area and compared to the opposite limb [18]. We conducted blood analysis and performed compression ultrasound on these women to confirm or rule out DVT and to identify the affected thrombotic vein.

We excluded pregnant women with other autoimmune diseases besides acquired/inherited thrombophilia, including malignancy, depression, genetic syndromes, and infections, or women undergoing any other treatments that could affect the results of serum evaluation. We also excluded women with other types of anemia, such as thalassemia, infection-induced anemia, genetic hemoglobin disorders, and any other hematological disorders.

### Statistical Analysis

Initially, the data were entered into Microsoft Excel for preprocessing. We checked and corrected any errors to have the exact real measured values. SPSS 24 was used for statistical analysis (IBM Corp. Released 2016. IBM SPSS Statistics for Windows, Version 24.0. Armonk, NY, USA: IBM Corp.).

The following measures were used to statistically describe the data: absolute and relative frequencies, average, confidence limits (95%) for mean, standard deviation, quartiles (Q1, Q2, Q3), and minimum and maximum.

We continued with univariate analysis, and here, we applied non-parametric tests to compare data sets. For categorical type, the Chi square or Fisher’s exact tests were used accordingly. For real data measurable on a ratio or interval scale, we used the Mann–Whitney test and the equivalent Wilcoxon test.

We also calculated the effect measured by risk in terms of odds ratio in both univariate (contingency 2 × 2 table analysis) and multivariate form (where we applied logistic regression).

For statistical decisions, we used a standard significance level of 5%.

## 3. Results

There were 59,021 births in the ten-year study period from 2015 to 2024. After applying the inclusion and exclusion criteria, we recruited 100 women with asymmetric lower limb edema in the puerperium. Among them, 67 (67%) cases were confirmed to have DVT in the puerperium, while the remaining 33 (33%) cases had negative peripheral thrombosis ultrasound assessment.

Women without thrombosis had a higher mean age (31.30 years) than women with thrombosis (27.43 years), *p* = 0.004 (Table 1).

### 3.1. Biological Parameters (Table 2)

- Platelet (PLT) count: the mean platelet count was significantly higher in the thrombosis group (280.78 × 10^9^/L) than in the non-thrombosis group (222.03 × 10^9^/L), *p* = 0.020.
- Neutrophil/lymphocyte ratio (NLR): women without thrombosis had a higher NLR (5.45) than women with thrombosis (4.73), *p* = 0.04.
- Neutrophil/platelet ratio (NPR): women without thrombosis had a higher NPR (0.040) than women with thrombosis (0.030), *p* = 0.011.
- Platelet/lymphocyte ratio (PLR): women in the puerperium with thrombosis had a higher PLR, although the difference did not reach statistical significance (*p* = 0.119).
- Mean platelet volume (MPV) was significantly elevated in the thrombosis group (9.88 fL) compared with the non-thrombosis group (8.33 fL), *p* = 0.001.
- Platelet distribution width (PDW) was significantly higher in the non-thrombosis group (18.92 fL) compared with the thrombosis group (14.29 fL), *p* < 0.001.
- D-dimer levels: women with thrombosis had significantly elevated D-dimer levels (6.16 μg/mL) compared with women without thrombosis (4.01 μg/mL), *p* = 0.020.

**Table 2:** Statistical significance of the demographic characteristics and clinical and biological parameters.

Parameter	Mann–Whitney U	Wilcoxon W	Z	Asymp. Sig. (2-Tailed)
Age (years)	713.000	2991.000	−2.881	0.004
PLT (×10^9^/L)	788.000	1349.000	−2.328	0.020
NLR	829.000	3107.000	−2.027	0.043
NPR	757.000	3035.000	−2.555	0.011
PLR	893.000	1454.000	−1.558	0.119
MPV (fL)	635.000	1196.000	−3.450	0.001
PDW (fL)	505.000	2783.000	−4.403	0.000
D-dimer (μg/mL)	735.000	1231.000	−2.321	0.020

Grouping variable: thrombosis.

### 3.2. Univariate Analysis (Table 3)

Using the Mann–Whitney test, all continuous variables analyzed revealed statistically significant differences between groups (*p* < 0.05), confirming biological parameter alterations in women with thrombosis. No significant associations were found between the mode of delivery, hypertensive disorders, low serum iron, and thrombophilia with thrombotic events, with *p*-values of 0.131, 0.434, 0.105, and 0.380, respectively. Anemia was significantly associated with thrombosis (*p* = 0.001). Women with anemia had an odds ratio (OR) of 5.77 for developing thrombosis.

A logistic regression model identified three significant predictors of thrombosis:

- MPV: a one-unit increase in MPV was associated with a 1.497-fold higher risk of thrombosis (*p* = 0.008).
- PDW: a one-unit decrease in PDW increased thrombosis risk (OR = 1.17, *p* = 0.003), suggesting a protective role for higher PDW.
- Anemia (hemoglobin value under 11 mg/dL): the presence of anemia increased the risk of thrombosis by 8.46 times (*p* = 0.003).
- Fibrinogen: a one-unit increase in fibrinogen levels increased the risk of thrombosis by 1.009-fold (*p* = 0.003).

**Table 3:** Statistical significance of thrombosis.

Evaluated Parameter	Type of Birth (Vaginal/C-Section)	Gestational Hypertension	Anemia	Lower Serum Iron Level	Thrombophilia	Fibrinogen
Asymptotic Significance (2-sided)	0.131	0.434	0.001	0.105	0.380	0.003
Odds Ratio	0.74	0.46	5.770	2.21	2.58	1.009

### 3.3. Multivariate Analysis

The multivariate analysis revealed that a one-unit increase in MPV led to a 1.497-fold increase in the risk of developing thrombosis. PDW seemed to be indirectly correlated with thrombosis, with a one-unit decrease resulting in a 1.17-fold increased risk of thrombosis. The presence of anemia in our patients increased the risk of developing thrombosis by 8.46-fold. Additionally, a one-unit increase in fibrinogen levels increased the risk of thrombosis by 1.003-fold.

## 4. Discussion

Due to the non-specific clinical presentation of DVT, its diagnosis is frequently delayed in clinical practice. The prevalence of DVT in our study was about 0.1135, aligning with studies that stated it to be between 0.025 and 0.1% [19,20]. While contrast venography remains the gold standard for confirming DVT, its invasive nature limits its widespread use. Additionally, venous compression ultrasound, though non-invasive, is less sensitive in iliofemoral thrombosis and less cost-effective in evaluating all cases with thrombotic suspicion [16,21,22,23,24]. In this study, we attempted to assess the detection rate of clinical lower limb asymmetric edema during the puerperium as a potential clinical sign of DVT. We also evaluated the associations between complete cell blood count (CBC) and D-dimer levels in these patients and the main obstetrical risk factors (gestational diabetes and hypertension, multiparity, cesarean section birth, lower limb varicose veins, and thrombophilia). We correlated our previously mentioned findings with the results of compression Doppler ultrasound evaluation of the venous system in these women. Our goal was to assess the sensitivity and specificity of the clinical method individually and in combination with blood analysis results and to verify our results with imaging techniques to detect DVT in the puerperium.

Correct clinical assessment of lower limb edema must establish whether the increased volume is an acute or chronic event, whether it is congenital or acquired, whether it is symmetrical or asymmetrical, and whether it is localized or generalized. Lower limb symmetrical edema is physiological and more pronounced during late pregnancy. When asymmetry arises, it is usually recognized as the main cause of DVT or lymphatic disease and, consequently, lymphedema. The physician must exclude lipedema, hematoma, dystrophy, idiopathic edema in women, and dermatological, infectious, or orthopedic causes [15,16,17,22]. In our analysis, two out of three women with asymmetric lower limb edema had confirmed DVT, suggesting that asymmetric lower limb edema might constitute a selection criterion for suspected peripheral thrombosis in pregnant and recently postpartum women, indicating those cases that need further Doppler evaluation.

Thrombotic events are recognized as the determining cause of impaired venous return associated with the slowing down of blood flow in the lower limbs, with consequent stasis [8,25]. Non-specific clinical DVT presentations vary in frequency as well as in severity between lower limb discomfort, edema of the whole leg or calf asymmetry, erythema, dilated collateral superficial veins, and calf discomfort during ankle dorsiflexion with the extended knee (Homan’s sign) [5,24]. In 2009, Chan et al. elaborated the acronym LEFt in an attempt to assess the risk of DVT through clinical examination (L—symptoms in the left lower extremity; E—edema: mid-calf circumference difference ≥ 2 cm; Ft—first-trimester presentation) [26]. In 2013, Righini et al. validated Chan et al.’s 2009 clinical score for assessing DVT in pregnancy [26,27].

Platelets (PLTs) are a well-known source of many angiogenic factors during normal pregnancy, being actively involved in spiral artery remodeling—in addition to their more widely recognized role in hemostasis—with thrombus formation [28,29,30]. Our results concur with those in the literature, with an elevated number of PLTs being significantly associated with thrombosis. The difference achieves more significance if we consider the high incidence of thrombocytopenia in normal physiological pregnancies through hemodilution or destruction [31]. Some of the investigated platelet complete cell blood count (CBC) indices that are correlated with thrombosis, besides their number (150–450 × 10^9^/L), are MPV, platelet distribution width (PDW), and platelet large-cell ratio (P-LCR) [32,33,34]. MPV reflects PLT dimensions, PDW assesses their variability in size, and P-LCR evaluates the proportion of circulating PLTs that are larger than 12 femtoliters (fL). Most studies in the literature have focused on platelet indices and DVT risk in orthopedic and cancer patients, with few addressing the correlation between these indices and the risk of DVT in the puerperium [34,35,36,37]. Studies have shown that, under normal conditions, MPV is inversely correlated with platelet number and increases with age. The MPV seems to increase in pregnancy; some studies suggest an association between increased MPV and unfavorable outcomes such as pre-eclampsia [38]. Study results are conflicting, perhaps due to differences in laboratory determination of MPV, with ethylenediaminetetraacetic acid (EDTA) exposure being temporally correlated with increased MPV, and due to indirect correlation with PLT counts [31,38,39]. Study findings vary regarding pathological conditions, particularly in the context of thrombotic event risk evaluation. However, most studies suggest that platelet count is positively correlated with DVT risk, as our results show. In contrast, PDW values seem to be inversely correlated with this risk [34,35,40]. The data we obtained differ from those mentioned above based on the indirect relationship between MPV and thrombotic risk, with MPV being statistically significant and directly correlated with thrombosis (a one-unit increase in MPV is correlated with an almost 1.5-fold increase in thrombotic risk), while PDW was not correlated with increased thrombotic risk [34,35,40]. The association between increased MPV and higher thrombotic risk can be explained by the fact that larger PLTs produce increased quantities of thromboxane, which affects the endothelium, leading to stronger PLT aggregation [41]. Whereas PDW seems to follow the trends reported in the literature, women in the puerperium without thrombosis have statistically significantly higher PDWs. All three platelet indices seem to facilitate the evaluation of DVT risk, with higher PLTs and MPVs and lower PDWs being associated with a higher likelihood of venous thrombosis in pregnancy. The immature platelet fraction (IPF) describes the proportion of newly released platelets into circulation. These young platelets are larger, are more reactive, and have a higher prothrombotic potential. In a thrombotic state, platelet consumption rises, due to clot formation, and stimulates the marrow to release more platelets, including immature ones, to compensate for this loss. Hayuningsih et al. in 2023 described IPF as a potential early indicator for pre-eclampsia [42]. Although the relationship between MPV and IPF is not always straightforward and linear, an important immature platelet fraction with an increased MPV release due to consumption might explain the alteration in CBC indices that we detected in these women, and perhaps, we can use these indices to detect women in the puerperium at risk of developing thrombotic events.

NLR, NPR, and PLR are cost-effective and easily accessible assessments. Studies suggest their usefulness across various pathologies, particularly in cardiovascular diseases; however, the results in the literature remain conflicting [43,44,45,46,47,48]. When elevated, they seem to be often associated with poor patient outcomes, reflecting systemic inflammation [44,49,50]. In pregnancy, these markers were evaluated for the risk of adverse outcomes linked to inflammation, risk of abortion, preterm birth, hypertension, or pre-eclampsia, but not for puerperium-associated thrombotic events [45,46,47]. To the best of our knowledge, this is the first study to analyze these three markers in the puerperium to assess the risk of thrombotic events. Our results indicate that in pregnant and recently postpartum women, NLR and NPR are statistically significantly higher in the non-thrombotic group (*p* = 0.043 and *p* = 0.011, respectively), while PLR is higher in women in the puerperium with thrombosis, although the difference did not reach statistical significance. The differences between these results and those in the literature might be due to the limited number of cases included or the physiological modifications of these parameters in pregnancy. Although our results might constitute a solid base for further research, they should be confirmed in a larger cohort to assess the utility of these markers in evaluating women in the puerperium with suspected thrombosis.

Most of the available clinical and paraclinical methods that help clinicians evaluate DVT-suspected patients seem to have limited applicability in pregnant and recently postpartum women. The clinical evaluation of pregnant women with suspected thromboembolic events is challenging due to physiological pregnancy-induced modifications, such as frequent but symmetrical inferior limb edema. The paraclinical evaluation of such cases, which usually confirms the clinically suspected diagnosis, creates more confusion in pregnant women due to altered normal-range blood parameters [51,52]. One such example is the fibrin-derived marker D-dimer. The evaluation of D-dimer levels is simple, accessible, and cheap. The literature is abundant with proof that, in the general non-pregnant population, low D-dimer levels coupled with a negative suspicion of DVT based on medical history and clinical evaluation are acceptably effective in ruling out the suspicion. However, this evaluation is scarcely explored in the puerperium due to the lack of associated D-dimer specificity, resulting from its 5- to 6-fold altered thresholds during gestation [23,53,54,55,56]. Morse et al. proved its proportional increasing trend during gestation [53]. In 2021, Sadeghi et al. proposed that a 1447 mg/L (mg/L) cut-off value for D-dimer had a 63.04% specificity, 87.50% sensitivity, and 98.3% negative predictive value for thrombotic events during pregnancy, regardless of gestational age [54]. However, in 2013, Wang et al. proposed thresholds for each trimester of pregnancy (660 mg/L, 2290 mg/L, and 3120 mg/L) [57]. Despite the debate on the cutoff value for D-dimer in pregnancy, our results confirm a statistically significant increase in the D-dimer value in pregnant and recently postpartum women with thrombosis [57]. The 2019 European Society of Cardiology guidelines state that D-dimer levels should be considered when ruling out pulmonary embolism in pregnancy [58].

Anemia and iron deficiency also seem to be associated with increased thrombotic risk in pregnancy, attributed partially to a reactive increase in platelet levels. Maternal anemia is defined by hemoglobin concentrations of less than 11 mg/dl, while iron deficiency is defined by serum iron levels of less than 50 micrograms/liter (μg/mL) [59,60,61]. It is an underestimated avoidable factor, and the exact mechanism remains to be established; however, the correlation finds support in the literature [62,63,64] and in the results of the current study. In accordance with most literature findings, but in opposition with Li et al. study results, we detected a statistically significant 8.46-fold higher risk of thrombosis in women in the puerperium with anemia, with low serum iron levels also seemingly linked to this pathology; however, the results did not reach the same statistical significance that was observed for lower hemoglobin levels [65,66].

Inherited and acquired thrombophilia are also linked to an increased risk of thrombotic events in the puerperium, constituting an important individual risk factor. Defects such as heterozygous factor V Leiden are associated with a 4–16-fold increased risk of thrombosis, with the risk reaching up to 40-fold if the defect is homozygous. Data in the literature on rarer thrombophilia, such as antithrombin and proteins S and C, describe annual incidences of 30–40% and 6–13%, respectively. The incidence of thrombotic events seems to be approximately 10% for the heterogeneous group of antiphospholipid antibodies [3,4,5,18,66]. Due to the low number of included cases—both asymmetric lower limb edema and puerperium thrombosis had low incidences due to the limited 10-year timeframe of this study—that were correlated with both acquired and inherited thrombophilia incidence in women in the puerperium with thrombosis, our data did not support any significant association between the two pathologies.

The results we obtained did not support any association between the modality of birth (vaginal or cesarean section) or pregnancy-induced hypertension and the risk of DVT. This may be due to the low molecular weight of heparin prophylaxis in all women with C-section births, the small number of included cases, and/or selection bias.

Our study limitations include the small number of cases analyzed due to the low prevalence of DVT, the 10-year study timeframe, and/or selection bias. The data must be interpreted cautiously due to geographical variations in risk factors. Finally, the lack of congruence between the results of our puerperium study and results reported in the literature, i.e., in terms of the modality of birth, pregnancy-induced hypertension, and thrombophilia being important DVT risk factors, needs to be carefully assessed in the context of the limited number of cases, which may have resulted in bias.

## 5. Conclusions

Significant lower limb inequality might be a useful criterion for high-suspicion DVT selection in the puerperium. The univariate and multivariate analyses revealed that MPV, fibrinogen, and anemia increase DVT risk, while PDW seems to be indirectly correlated with peripheral thrombotic risk.

## Figures and Tables

**Table 1:** The demographic characteristics and clinical and biological parameters of our included cases.

Parameter	Thrombosis	Mean	95.0% Lower CL for Mean	95.0% Upper CL for Mean	StDev.	Percentile 25	Median	Percentile 75	Minimum	Maximum
Age (years)	No	31.30	29.14	33.46	6.09	28	33	36	17	40
	Yes	27.43	25.91	28.96	6.26	23	27	32	17	41
PLT × 10^9^/liter (L)	No	222.03	196.65	247.41	71.57	174	205	247	113	456
	Yes	280.78	251.10	310.46	121.68	187	256	346	132	678
NLR	No	5.45	4.49	6.42	2.71	4.04	4.97	6.37	1.71	16
	Yes	4.73	3.84	5.61	3.64	2.45	3.83	6.20	0.37	25.39
NPR	No	0.040	0.032	0.049	0.025	0.027	0.037	0.046	0.016	0.144
	Yes	0.030	0.025	0.036	0.020	0.016	0.025	0.040	0.002	0.092
PLR	No	142.8372	125.8150	159.8595	48.00614	105.89	132.6087	169.15	86.24	292.14
	Yes	166.8591	147.8937	185.8246	77.75293	114.85	149.3023	196.75	57.03	524.79
MPV femtoliter (fL)	No	8.33	7.62	9.04	2	6.50	7.70	10	5.20	12.10
	Yes	9.88	9.49	10.27	1.60	9.40	10	10.90	4.90	13.10
PDW (fL)	No	18.92	17.23	20.60	4.75	16.60	18	23.50	11.20	26.30
	Yes	14.29	13.25	15.33	4.26	11.40	12.70	16.50	9.20	27.30
D-dimer microgram/milliliter (μg/mL)	No	4.01	3.19	4.83	2.25	2.09	4.70	5.30	0.19	10.47
	Yes	6.16	4.34	7.98	7.45	4.30	4.99	6.10	0	61

## Data Availability

The data used to support the findings of this study are available upon request to the corresponding author.

## Abbreviations

The following abbreviations are used in this manuscript:

CBC	Complete cell blood count
DVT	Deep vein thrombosis
EDTA	Ethylenediaminetetraacetic acid
MPV	Mean platelet volume
NLR	Neutrophil–lymphocyte ratio
NPR	Neutrophil–platelet ratio
OR	Odds ratio
PLT	Platelet
PDW	Platelet distribution width
PLR	Platelet–lymphocyte ratio
VTE	Venous thromboembolism
